# Breastfeeding practices of ethnic Indian immigrant women in Melbourne, Australia

**DOI:** 10.1186/1746-4358-8-17

**Published:** 2013-12-18

**Authors:** Natasha Maharaj, Mridula Bandyopadhyay

**Affiliations:** 1 Judith Lumley Centre, La Trobe University, 215 Franklin St, Melbourne, Australia; 2 College of Health & Biomedicine, Victoria University, McKechnie Street, St Albans, Victoria, Australia

**Keywords:** Breastfeeding, Acculturation, Cultural identity, Indian immigrants, Australia

## Abstract

**Background:**

The health benefits of breastfeeding are well documented in public health and medical literature worldwide. Despite this, global rates of breastfeeding steadily decline during the first couple of months postpartum. Although immigrant women have higher initiation rates and a longer duration of breastfeeding overall, breastfeeding practices are compromised because of a myriad of socioeconomic and cultural factors, including the acculturation process. The objective of this study was to show how acculturation and cultural identity influenced breastfeeding practices among Indian immigrants in Melbourne, Australia.

**Methods:**

Twelve case studies were employed to gather narratives of women’s lived experiences. Ethnographic field research methods were used to collect data, including participant observation, semi-structured interviews, case studies, and life histories. This provided in-depth information from women on various aspects of the immigrant experience of motherhood, including infant care and feeding. Participants were opportunistically recruited from Indian obstetricians and gynaecologists. Women identifying as ethnic Indian and in their third trimester of pregnancy were recruited. Interviews were conducted in women’s homes in metropolitan Melbourne over a 12 month period between 2004 and 2005. Data were coded and analysed thematically.

**Results:**

All women identified as ethnic Indian and initiated breastfeeding in accordance with their cultural identity. Social support and cultural connectivity impacted positively on duration of breastfeeding. However, acculturation (adopting Australian cultural values and gender norms, including returning to paid employment) negatively influenced breastfeeding duration. In addition, the high reliance of recent immigrants on the advice of healthcare professionals who gave inconsistent advice negatively affected exclusive breastfeeding.

**Conclusions:**

For ethnic Indian immigrant women breastfeeding practice is closely linked to acculturation and identity construction, both personal and communal. The lack of social and cultural networks for recent immigrants prevents their involvement in the cultural systems that traditionally support breastfeeding. With this in mind, healthcare professionals should deliver services in a culturally appropriate and sensitive manner where women feel supported as well as empowered.

## Background

The health benefits of breastfeeding and exclusive breastfeeding are substantial both for the mother and the infant. This has been well documented in public health and medical literature worldwide [1,2]. Breast milk is the most complete and wholesome nutrition for a newborn. It is also cost-effective, disease-preventing and health-promoting [3]. Despite various advantages of breastfeeding, rates of breastfeeding globally decline rapidly in the first four to eight weeks postpartum [4,5]. Many women either do not breastfeed or breastfeed for only a short duration, or start breastfeeding late [6]. Factors known to affect breastfeeding practice include socioeconomic status, maternal education and employment, prenatal intention, maternal attitude and confidence, ethnicity, a shift from a rural to an urban environment (residence) and to nuclear families, emulating Western lifestyles, the influence of healthcare professionals and the availability of infant formula [6,7]. Most women who discontinue breastfeeding early do so because of perceived difficulties such as lack of milk, unsuccessful breast attachment, their baby being unable to suckle, leakage, pain and so on [2]. Many hospitals in Australia have adopted the United Nations Children’s Fund and World Health Organization’s 1992 Baby Friendly Hospital Initiative and health care professionals are trained to support and promote breastfeeding.

High breastfeeding initiation rates and long duration of breastfeeding is still the norm in many developing countries. However, exclusive breastfeeding is rare in some of these countries because of cultural practices associated with lactation and breastfeeding (withholding colostrum, giving prelacteal feeds) [4,8]. Therefore, when women from such societies migrate - rural to urban within a country or to another country - their breastfeeding practices including initiation and duration are affected. This may be due to the process of acculturation, that is, adapting to a new environment. Earlier studies indicate an association between the degree and level of acculturation and breastfeeding practices and behaviours. This means that breastfeeding initiation is highest among women who are the least acculturated and lowest among women most acculturated. For example, a shorter length of residence was found to be positively related with breastfeeding initiation and duration in the United States among Latino and South Asian immigrants [9-11], and longer length of residence in the United States was found to negatively influence breastfeeding initiation and duration [12]. Likewise, this trend was observed among immigrants from Vietnam in Australia [13]. By contrast, a recent study among Vietnamese immigrants to Canada states that decision to bottle feed was not related to acculturation but to conflicts between Vietnamese cultural practices and the configuration of the social space in Canada [14].

Studies have consistently shown that immigrant women from developing countries have higher initiation rates and longer duration of breastfeeding compared to women in their host countries [10,12,13]. This is generally attributable to the cultural norms relating to breastfeeding practices prevalent in their countries of origin. However, there is dearth of evidence on how construction of cultural identity influences breastfeeding practices among recent and established immigrants, particularly, Indian immigrants in Australia.

### Ethnic immigrant Indians and breastfeeding

Breastfeeding is universal and prolonged in India [15-17] – both in urban and rural areas – and continues into early childhood years [18-20], but initiation is delayed because of the perception that mother’s milk flows two to three days after childbirth [21]. Indian mothers do not exclusively breastfeed in India due to traditional practices; the exclusive breastfeeding rate in India was 46% at less than six months in 2005–2006 [22]. The varied cultural practices associated with lactation and breastfeeding in India include anything from exclusive feeding to varied amounts of prelacteal and supplemental feeding with infant formula and other types of animal milk [8].

Not much is known about this particular group of women’s breastfeeding practices in Australia, although Indian ancestry immigrants constitute two per cent of the Australian population [23] and this percentage is likely to increase in the near future. In the last few decades, there has been a significant increase in the number of ethnic Indians migrating to Australia. Victoria has the largest group of ethnic Indian residents in Australia. Melbourne has a heterogeneous population of Indians with regard to religion, language, and place of origin. They are one of the larger new immigrant populations and comprise peoples from India as well as Fiji, South Africa and the United Kingdom amongst other Indian groups. Despite this, there is limited literature on Indian immigrant experiences in Australia, hence, very little is known about their culture and their needs in Australia. Still less is known about the way they acculturate to their new environment, in particular, the way in which they adapt to parenting. Although research on Indians settling in different Western societies has been documented in acculturation literature, there is no evidence on how acculturation and identity impacts breastfeeding practice amongst Indians in Australia. Therefore we explored one aspect of parenting in depth and examined how cultural identity construction influenced breastfeeding initiation and duration among ethnic Indian immigrants in Melbourne.

## Methods

The general aim of this study was to investigate the influence of culture and identity on the immigrant experience of early motherhood among ethnic Indian women living in Melbourne. This paper specifically discusses breastfeeding and the wider cultural and social aspects impacting its practice amongst this group of women.

Ethnographic field research was employed to gather an in-depth understanding of the complex social, cultural and traditional construct of identity and its influence on breastfeeding. Case studies were used to capture the personal lived experiences of individual women and their families to illustrate the complex narratives and holistic nature of the experiences of cross-cultural parenting. This provided insight on the broad themes of acculturation and identity construction, cultural values towards childrearing and traditional practices surrounding mother and infant care, including breastfeeding. Fieldwork for the study was conducted between 2004 and 2005. The Human Research Ethics Committee of the University of Melbourne approved this project (Reference 43/2003).

### Study participants

Ethnic Indian women born in India, Fiji, the United Kingdom and South Africa were recruited for this study. They were all first generation immigrants to Australia. Some were established immigrants having migrated as children with their families and others had migrated as adults for economic reasons between five and 10 years prior to the study. Of the recently arrived women, many had migrated with their husbands or to marry resident Indians in Australia. Women who met the following criteria were recruited in the study: in the third trimester of pregnancy and identified as an ethnic Indian immigrant. During the course of this study participants were expecting a child, gave birth and introduced to solids. All were from middle-class backgrounds; the majority had a tertiary qualification and about half were in paid employment. Secondary participants were members of the women’s family who were present in the home during data collection and who wanted to participate in discussions and gave consent to be tape-recorded. Women have been given pseudonyms when quoted.

### Study site

The study was conducted in metropolitan Melbourne, specifically areas in north-western, north-eastern, and south-eastern suburbs of the city as the Indian population is concentrated in these areas. The interviewing and observation of primary and secondary participants was conducted in women’s homes by NM.

### Data collection

Opportunistic sampling occurred with the assistance of six Indian obstetricians and gynaecologists practicing in metropolitan Melbourne. As this was part of a larger project, because of convenience and timeliness, only Indian doctors were targeted. Ethnicity was not recorded in public hospitals during the study period, hence sampling and recruitment was limited to Indian doctors for maximum exposure. Twelve case studies were collected using ethnographic methodology including participant observation and in-depth interviews with women and their families, in their homes, over a 12 month period. The broad themes of immigration, acculturation, identity, and the cultural context of mother and infant care and child rearing were explored. Data collection instruments included face-to-face in-depth interviews, case-studies, participant observation and life histories.

For each individual case study, a series of in-depth, semi-structured interviews and unstructured/informal conversations with the woman were conducted over a period of time. Interviews were conducted in English as all women were fluent in English. All interviews were audio-taped with the participant’s consent and later transcribed for accuracy of information. Observations were also noted immediately after visits with the women.

Mothers were followed for approximately 12 months, from before they gave birth to when they began introducing their infant to solid foods, and beyond. Information on women’s intentions to breastfeed was collected before birth and information about their actual practices was collected after birth. Due to the explorative and iterative nature of qualitative research, new themes continued to emerge throughout the interview process, and each successive interview was initiated with these themes in a semi-structured manner to facilitate open-ended discussion. These themes were also validated by the participants at the beginning of each visit. A semi-structured interview guideline was developed and this was adapted after each interview to accommodate emerging themes. Data collection continued until data saturation was reached, that is, until no new themes emerged.

### Data analysis

Data analysis was conducted using a general inductive approach [24]. The interview transcripts were analysed through multiple readings developing themes or categories. Broad or general categories were derived from the study aim and specific themes from the data objectives. These resulted in summarising categories that enabled the identification of themes, which were most relevant to the research objectives. Finally, frequent, dominant, or significant themes observed in raw data were identified in all of the case studies; divergent experiences were also accounted for in the analysis, that is, divergent views that emerged during analysis lead to further sampling to check whether the phenomenon was atypical, ensuring that diversity of experience was transparent in the findings. Themes were then crosschecked with participants for accuracy and verification of data gathered in previous interviews in order to maintain trustworthiness of the data, and to prevent researcher bias in the data analysis. Sampling continued until no new themes emerged, that is data collection ceased when the same themes were being reiterated.

## Results

Women in this study were from urban areas in India, Fiji, the United Kingdom and South Africa. Most had tertiary education and half were involved in paid employment. They were between the ages of 26 and 42 at recruitment and first interview, with most being in their late 20s. All were married and expecting a child when first interviewed, with seven of the 12 women having had other children. Five women were recently arrived immigrants; another five had immigrated between five and 15 years prior to interview and two had immigrated as children. Most women immigrated after getting married to men who were already resident in Australia.

All women identified as ethnic Indian, had pride in their cultural heritage, and had intentions of initiating breastfeeding in accordance with this ethnic and cultural identity. Motherhood is highly valued in Indian culture and women are defined, communally and personally, by their role as mothers. Breastfeeding is an integral aspect of mothering and is, consequently, seen as natural and normal. Therefore, unsurprisingly, all women in this study initiated breastfeeding. However, their acculturation and sense of identity - cultural and gendered - played an important role in the maintenance and duration of breastfeeding. This was seen through factors such as employment status, cultural connections and social support as well as advice from health professionals that influenced women’s breastfeeding practice.

### Knowledge of breastfeeding

Overall, women were knowledgeable about breastfeeding. This knowledge came from either their cultural and traditional practices – as taught by their mother or mother-in-law or by observing what female relatives did in their home country – or from professional recommendations in Australia. Women were aware that traditionally the recommendation was to breastfeed for as long as possible as it is ‘healthy’ for the baby. Additionally, they were aware of the practice of demand feeding and anticipated following this practice in Australia. They were also aware that Australian health professionals recommended exclusive breastfeeding for six months, and thereafter to maintain breastfeeding for at least a year.

### Prenatal intentions to breastfeed vs actual initiation and duration of breastfeeding

Women’s intentions to breastfeed were inexorably tied to their cultural identity and heritage. During antenatal interviews their intention to breastfeed was clear and steadfast. All women had intention to breastfeed, as ‘this is what Indian women do as mothers’, and were intent on breastfeeding for a minimum of six to 12 months.

However, after childbirth women’s practice differed from their initial intention. All women initiated breastfeeding immediately after birth as instructed by their midwives. However, when women felt that they experienced breastfeeding difficulties, infant formula was introduced. All but one woman gave infant formula during the course of breastfeeding and most began infant formula feeding before six months of age, with five starting at hospital*.* Duration of breastfeeding declined with several women breastfeeding for a far shorter duration than originally intended and few breastfeeding for 12 months. Usually breastfeeding was abandoned earlier than intended because of the confusing messages given by health professionals. Some women said they were asked to follow a feeding schedule and not to demand feed, which undermined their traditional approach to breastfeeding thus affecting their confidence in their ability to care for their baby.

Health professionals seemed to have a significant influence on women’s decisions regarding supplemental feeding, which influenced their breastfeeding practice. This was more apparent amongst the recently arrived women who looked to health professionals for guidance in appropriate healthcare and for what is ‘right’ in Australia.

My mother told me that it is very good to breastfeed, but according to the advice of the doctors here, I started him [baby] on formula. My mother insisted that it is good to have breast milk. Yes, I think sometimes he [baby] is not satisfied with my milk because he needs something more. I am not saying that my breast milk is not coming, maybe his capacity is more. The maternal and child health nurse/midwife told me to give him one or two bottles a day, but also to continue with my breast milk. (Preeti)

Preeti knew that breastfeeding is the traditional way of feeding and is best for the baby but like other recently arrived women, she was influenced by her health professional to start supplementary feeding. Women like Preeti were easily swayed by health professionals whom they felt ‘know best’ and could advise upon the ‘right’ way of doing things in Australia.

### Major factors impacting on breastfeeding

#### Paid employment

Women in paid employment felt that they had to cease breastfeeding before resuming work. Therefore, women on maternity leave started weaning their infants prematurely even though they had intended to breastfeed for longer prenatally. These women wanted their babies to get accustomed to bottle feeding. Expressing breast milk was seen as time consuming and inconvenient (also this is not practiced in their home country and is unfamiliar) so breast milk was substituted with infant formula (which is easily available) when women were at work, and breastfeeding eventually ceased:

Breastfeeding, in India it is a must. If you don’t you are sort of looked down upon like you are not doing the right thing as a mother. Because I fed Leila [when at home] till she was about 10 months, and we went to India when she was about 14 months, and back there people are still breastfeeding their babies till they are past a year old, so you had to answer to them. ‘Why aren’t you feeding her? You should still be feeding her!’, and I said, ‘No, I can’t feed her because I am back to work now’. (Nina)

Nina had migrated from India over ten years prior to the study and felt that she was acculturating to an Australian way of life. For Nina, gaining a sense of belonging here meant adopting Australian gender norms. As well as needing to work to maintain a good standard of living, she wanted to work as she felt this made her independent and gave her an identity apart from ‘mother’. She saw herself as a Westernised Indian and felt that this set her apart from recently arrived Indian women, such as her sister-in-law, whose adoption of the traditional gender role for Indian mothers did not accommodate working outside the home.

Nina was on maternity leave when she had her baby and knew that she had to return to work in a few months, so she began weaning in preparation. She was educated and understood the benefits of breast milk but also felt that expressing took up a lot of time and effort when she had to balance duties at home and work.

Employed women stated that they were aware of the benefits of exclusive breastfeeding for six months and continuing breastfeeding beyond. Also, they knew that this is what Indian mothers do, and that it is highly recommended by Australian health professionals. However, they felt that to prosper in Australia both husband and wife had to work. Also, the values of this society meant that women needed to embrace roles other than ‘mother’ to feel accomplished. Acculturating women were dealing with a shift in personal identity from the traditional Indian mother to incorporating a more Westernised gender roles. This impacted on their breastfeeding behaviour and practice.

#### Advice of healthcare professionals

Recently arrived women relied on the advice of health professionals for the ‘right’ approach to breastfeeding in a culture that was new to them. Most women had positive overall experiences with health professionals. However, some women reported confusion and stress surrounding breastfeeding when they were shown more than one position to breastfeed in, when they were told about feeding schedules as well as demand feeding and when they were offered infant formula when unsure about the adequacy of their milk supply:

They [midwives] said ‘three hours, after that, five hours’, that’s it. They [midwives] didn’t mention anything like, ‘sometimes the baby feels like breastfeeding’, they didn’t tell me anything about that, but my Mum told me ‘If she feels like taking breast milk, just give her, don’t hesitate to give her whenever she feels like it’. . . In the hospital they [midwives] used to give formula, because maybe I didn’t have enough milk. (Rohini)

Rohini had recently arrived in Australia and had been living in Melbourne for just two years. She was unemployed and her husband worked long hours. She had no family or other social networks. She said that she felt lonely and missed her family, more so after childbirth with no friends or community support. This impacted on breastfeeding especially when she felt that she did not have enough milk. She knew that according to her culture she should breastfeed her child as she had seen other women in her family do, but felt that this was difficult without support and encouragement. She spoke to her mother in India on the phone for reassurance but could not help being influenced by Australian health professionals whom she thought ‘know best’.

Support is integral in the Indian conceptualisation of motherhood. Senior extended family members such as mothers, mothers-in-law and sisters-in-law are often present during the postpartum to assist new mothers. Their advice and opinions are highly valued and relied upon, and their role is central to infant care. They are also role models and not having them in close proximity after childbirth robs women of guidance and support.

#### Cultural isolation and lack of social support

Recently arrived immigrant women who were not employed and who did not have any extended family here felt ‘lonely’ and ‘out of place’ after childbirth. In India, hospitals allow the practice of ‘rooming-in’ where a female family member is allowed to stay overnight with the new mother to assist her with caring for the baby and initiating breastfeeding. There is also a confinement period during which mothers and mothers-in-law take care of the new mother and baby and support breastfeeding, once they return home. Since these cultural systems are not in place in Australia, women felt they could not have the level of rest and assistance at hospital or at home that they would have liked to facilitate successful initiation and maintenance of breastfeeding. Most extended family did not live in Australia and visited for short periods of time, leaving many women depending on their husband’s support:

When Rishaan was in hospital, I think it was the second or third night, he got really hungry obviously. And this night I started crying because Kiran [husband] was not there at that time, and I didn’t know what to do and there was no milk coming out. And because I was stressed out the nurses just said to me, ‘look, it is up to you if you want to give him formula’, and I just said ‘yup, that is it, do it’. (Ria)

Ria who migrated from Fiji over five years prior to the study and did not have her own family around after the birth of her baby to engage her in the customs and traditions that support breastfeeding, relied on her husband for encouragement. However, he was at work during the day and the absence of elder women meant that Ria felt overwhelmed and unsure of herself at this vulnerable time. Difficulties with breastfeeding - her child did not attach to the breast and she felt that she had insufficient milk - compounded this lack of familial support and lack of confidence, impacting negatively on breastfeeding.

For women with no extended family support, husbands and health professionals become important in the decisions surrounding infant care. Health professionals, due to their education and expertise in this area, as well as their positions of authority, are looked upon, often unquestioningly, for advice and guidance.

In contrast, breastfeeding practice was positively impacted when women felt culturally connected and socially supported:

I will do it [breastfeed] anywhere, I have never had any issues…all the time, wherever they needed it, I just did it…she [her mother] was exactly the same, Mum never worried about breastfeeding anywhere (Patricia).

Patricia migrated to Australia as a child with her family and married an immigrant man who had also migrated to Melbourne as a child. She knew that she wanted to breastfeed her babies but unlike the newly arrived women in this study, she was well established in Australia, had community networks and support systems in place and had more confidence in her ability to navigate motherhood. She said that breastfeeding was something that her mother did and that she was going to do the same. Patricia’s mother, after whom she was modelling her mothering approach, was also in Melbourne and Patricia felt confident in her dual Indian-Australian identity. The interviewer observed Patricia breastfeed on demand on many occasions, in keeping with the traditional way of feeding, without regard for scheduling feeds. Patricia exclusively breastfed all her children and ceased breastfeeding each child only when she became pregnant again. Her confidence in what she knew to be best for her babies seemed to be fuelled by having her role model (mother) present and by her sense of belonging in Australia. She did not feel that she had to defer to what others were advising as she was informed and secure in her own beliefs.

Unlike acculturating women, who were still trying to cement their belonging in Melbourne and wanted to fit into the prevailing cultural system, Patricia grew up in Australia and felt that she had nothing to prove by conforming to Australian gender roles. She also strongly identified as Indian, communally as well as personally, and prioritised her role as ‘mother’, wanting to mimic the Indian way of mothering that was passed down to her by her mother, which included totally devoting herself to the care and raising of her children for the time being. Patricia had strong opinions on what she saw as being the positives of Indian motherhood and was proud of asserting these practices and values. She was also able to stay at home to care for her children without the pressure of having to return to paid employment, was involved in her cultural community and had the daily support of her family (husband, mother and father) for whom breastfeeding was a normal and valued practice.

For the majority of women in this study, when mothers and mothers-in-law were not around in the postpartum period, women depended on their husbands to reassure them in their efforts to breastfeed. Those who had their husband’s encouragement, and were well supported by extended family and other social networks and felt culturally connected were able to persist in their attempts at breastfeeding despite difficulties such as problems with attachment and soreness of nipples. By contrast, those who were introduced to infant formula at hospital, whose husbands viewed infant formula feeding as a good option and had no family or community networks, felt comfortable supplementing with infant formula when breastfeeding seemed difficult or inconvenient. Since their midwives had offered this option it seemed like an acceptable and comparable method of feeding to women who were culturally and socially isolated and heavily dependent on the advice of health professionals.

## Discussion

In keeping with the Indian culture and tradition, women in this study were aware of the benefits of breastfeeding [25,26]. This study confirms the overall high initiation rates of breastfeeding (putting child to breast immediately after birth and attempting to feed colostrum) among Indian immigrants in Australia as evidenced in other studies of Indians immigrants [10,12]. Regrettably, it also reveals the attrition of exclusive breastfeeding and a marked reduction in the duration of overall breastfeeding. Socioeconomic and cultural factors such as resumption of paid employment, lack of maternity leave, social networks, family support, and cultural isolation and loneliness are some of the main factors affecting breastfeeding.

Recently arrived women relied on midwives and maternal and child health nurses for the ‘right’ advice on breastfeeding in Australia. Unfortunately women experienced distress and confusion when they were told about both scheduling feeding and demand feeding. Women were also shown different feeding positions by different health professionals that led them to feel that the previous position they were trying was not working; this undermined their confidence in their ability to breastfeed. Offering women infant formula when they were uncertain about their breast milk supply compounded this effect as it caused them to believe that they had inadequate breast milk. Through poor practice, health professionals contributed significantly in eroding traditional breastfeeding practices for the women in this study.

In India, health professionals are highly regarded for their knowledge and position, and therefore immigrant women in this study relied on Australian health professionals’ advice unquestionably. When infants were introduced to infant formula at the hospital, women felt it was acceptable to continue infant formula feeding. The unequal power dynamic present between health professionals’ and immigrant women, was clearly apparent in this study, as some women uttered sentiments of ‘they know best’. This is a common phenomenon in a medical professional-patient relationship [27], and can be particularly pronounced where women are in a culture that is unfamiliar to them, and come from a culture that is even more hegemonic like the caste/class system in India. Women depended on their health professionals for good practice, medically and culturally, in Australia. It is therefore essential that health professionals who assist new mothers be consistently educated in the best ways to support and maintain exclusive breastfeeding.

The physiology of breastfeeding as a reproductive matter is not talked about openly in Indian society. This knowledge is passed on to young mothers by elder female relatives in the postpartum period. Young mothers generally share their mothering responsibilities with older mothers, usually their mother, mother-in-law and sisters-in-law [28]. Lack of this social connection and community support in Australia made women more reliant on health professionals’ advice. Further, the absence of their cultural systems in Australia meant an interruption in their rites of passage into motherhood, generally, and breastfeeding, specifically.

Recently arrived and less acculturated women can feel great stress when confronted with models of healthcare that are foreign to them [29]. Women in this study were balancing two distinct models or cultural approaches to motherhood: the Western or Australian model (individualistic or self-sufficient approach) in which women are generally expected to cope on their own and the traditional or South Asian approach to mothering which is dominated by a cultural expectation that the new mother be supported and nurtured. This independence versus reliance underpins the divide between these two approaches and that of the expectations of health service providers and immigrant mothers. In looking to service providers, women need to feel that their beliefs and customs are being understood and appreciated.

In India, the role of ‘mother’ is highly valued and women prioritise this once they have a baby [30]. The economics of women’s daily lives, particularly after childbirth in Australia, discouraged them from doing what they knew to be best for their babies. Women involved in work outside the home had to balance this with other household duties, rendering mothering activities such as expressing breast milk a low priority, when there were other more time-efficient methods of feeding their babies, for example with infant formula. Further, lack of support from family and confusing advice from health professionals acted in tandem impacting breastfeeding practices. Besides, women who returned to work after childbirth were also acculturating which meant reconstructing their personal or gendered identities in accordance with Australian cultural values and norms.

### Limitations of the study

One limitation of the study is the relative economic homogeneity of the study sample as all women were middle class and educated and came to Australia through skilled migration or as a result of marrying someone who came to Australia through skilled migration; although the study sample includes women who were born in India, Fiji, the United Kingdom and South Africa, and women who were newly arrived, established, and had been here since childhood. Not all segments of the Indian community could be included, therefore, findings may not be generalisable to other socio-economic sub-groups of immigrants from South Asia. However, it is the depth of information gathered, and not the breadth or statistical representation that is important in an ethnographic study; hence, this has not compromised the credibility of findings from this research.

### Implication for practice

The cultural approach of immigrant women needs to be understood and remain at the foreground of any discussions on mother and infant care and feeding. However, we also need to bear in mind the cultural variability of ethnic Indian women due to the diasporic nature of the Indian population worldwide, the various birth places and differing levels of exposure to Western culture, and to assess a woman’s acculturation before assuming her understanding of Western models of childcare and rearing.

Support is a cultural expectation amongst Indians, where family and community (elder familial females traditionally and husbands and ethnic community in Australia) support and promote breastfeeding through traditional aspects of care during the postpartum period. In the case of recently arrived women lacking family and established community networks, ethnically specific peer support could be introduced at hospital to assist with the cultural aspects of breastfeeding. This could be followed through at women’s homes after discharge. This could help both Indian women and provide a possible model for intervention for other immigrant women in Australia.

In understanding women’s experiences and decisions regarding breastfeeding, the social and cultural context should be taken into account. In the case of Indian immigrants, the extended family, such as mothers and mothers-in-law who have an integral role traditionally, husbands who have an active role in Australia and health professionals who are looked upon as experts are all critically important to breastfeeding. It is therefore essential that health professionals who come into contact with new mothers are knowledgeable about the benefits of exclusive breastfeeding and about how to best encourage and support breastfeeding mothers to continue exclusive breastfeeding.

## Conclusions

No matter where their place of birth, the Indian immigrant women in this study contextualised the birth of their children with a culture of ceremony and celebration placing great importance on the customs and traditions surrounding motherhood. However, for recent immigrants, circumstances beyond their control prohibited re-creating customs and traditions relating to childbirth, particularly, breastfeeding. Since women in this study were of an educated strata of society we hypothesise that these circumstances would be heightened for Indian immigrant women who might be from disadvantaged socioeconomic backgrounds. Therefore, health professionals should deliver services in a culturally appropriate and sensitive manner, where women feel they are understood and valued for their way of doing things. This could empower women with a sense of pride in their ethnic identity and belonging in this country as well as cater to their psychological and emotional wellbeing.

## Competing interests

The authors declare that they have no competing interests.

## Authors’ contributions

NM carried out the conceptualisation, design, data collection and analysis for the study. NM and MB contributed to the interpretation of the findings and the drafting of the article. Both authors read and approved the final manuscript.
